# Do Lactation-Induced Changes in Ghrelin, Glucagon-Like Peptide-1, and Peptide YY Influence Appetite and Body Weight Regulation during the First Postpartum Year?

**DOI:** 10.1155/2016/7532926

**Published:** 2016-05-23

**Authors:** D. Enette Larson-Meyer, Jessica Schueler, Erin Kyle, Kathleen J. Austin, Ann Marie Hart, Brenda M. Alexander

**Affiliations:** ^1^Department of Family and Consumer Sciences (Human Nutrition), University of Wyoming, Laramie, WY 82071, USA; ^2^St. Charles Medical Center, Bend, OR 97701, USA; ^3^Department of Campus Recreation, University of Wyoming, Laramie, WY 82071, USA; ^4^Department of Animal Science, University of Wyoming, Laramie, WY 82071, USA; ^5^School of Nursing, University of Wyoming, Laramie, WY 82071, USA

## Abstract

To determine whether fasting and meal-induced appetite-regulating hormones are altered during lactation and associated with body weight retention after childbearing, we studied 24 exclusively breastfeeding women (BMI = 25.2 ± 3.6 kg/m^2^) at 4-5 weeks postpartum and 20 never-pregnant controls (BMI = 24.0 ± 3.1 kg/m^2^). Ghrelin, PYY, GLP-1, and appetite ratings were measured before/and 150 minutes after a standardized breakfast and 60 minutes after an* ad libitum *lunch. Body weight/composition were measured at 6 and 12 months. Fasting and area under-the-curve responses for appetite-regulating hormones did not differ between lactating and control groups; ghrelin_acyl_, however, tended to track higher after the standardized breakfast in lactating women and was higher (*p* < 0.05) after the* ad libitum* lunch despite a 24% higher energy intake (*p* < 0.05). By 12 months, lactating women lost 5.3 ± 2.2 kg (*n* = 18), whereas control women (*n* = 15) remained weight stable (*p* = 0.019); fifteen of the lactating women returned to within ±2.0 kg of prepregnancy weight but three retained >6.0 kg. The retainers had greater (*p* < 0.05) postmeal ghrelin rebound responses following breakfast. Overall these studies do not support the hypothesis that appetite-regulating hormones are altered during lactation and associated with postpartum weight retention. Altered ghrelin responses, however, deserve further exploration.

## 1. Introduction

Epidemiological studies suggest childbearing is an important contributor to development of obesity in many women [1–7]. Weight retention associated with pregnancy is estimated to be between 0.5 and 3.8 kg at 2.5 years postpartum [1, 2, 4–10]. According to several long-term US studies, including the National Maternal and Infant Health Survey [11] and the First National Health and Nutrition Examination Survey (NHANES) [1], average weight gain associated with one live birth is approximately 1.6 to 1.7 kg above age-associated weight gain. Higher than average permanent weight retention, however, is experienced by many women who become overweight or obese following the birth of a single child. For example, according to NHANES, having one live birth increases the risk of becoming moderately overweight (body mass index, BMI > 27.3 kg/m^2^) by 60% and becoming obese (BMI > 30.0 kg/m^2^) by 110% in Caucasian women [1].

Weight loss at 6 months postpartum has emerged as a key predictor of long-term weight retention [1, 4], highlighting the importance of early postpartum weight reduction. In one long-term study of 795 women, those who lost all pregnancy-associated weight gain by 6 months postpartum were 2.4 kg heavier eight to ten years later, whereas those who retained weight were 8.3 kg heavier [4]. Even though breastfeeding often emerges as a protective factor against weight retention [4, 12, 13], supporting research is inconsistent [14, 15]. In fact, Butte and Hopkinson [14] observed that the effect of lactation on postpartum weight reduction is highly variable and may even contribute to net weight gain in some mothers.

Although socioeconomic, environmental, and lifestyle factors, including number of children, maternal age, and exercise habits, are important determinants of weight retention following pregnancy, it is possible that hormonal changes that occur as a result of pregnancy or lactation may also be at play. Recent evidence has brought attention to the gut hormones as key contributors to the regulation of food intake and energy balance [16–19]. These hormones include the stomach-derived orexigenic peptide ghrelin and the intestinal-derived anorexigenic peptides glucagon-like peptide-1 (GLP-1) and peptide YY (PYY), which serve as signals to the appetite-regulating centers of the brain. It is not known, however, whether ghrelin, PYY, or GLP-1 (or other modulators of the brain-gut axis) is altered in the postpartum state, particularly during lactation. A few human studies have measured circulating ghrelin in postpartum women [20–24]; however, it remains unclear if and how these hormones fluctuate across the first postpartum year, both during fasting and in response to a meal, and whether circulating concentrations influence postpartum weight retention.

The primary purpose of this study is to determine whether the appetite-regulating hormones ghrelin, PYY, and GLP-1 are altered during lactation (both fasting and in response to a meal) and to determine whether circulating concentrations of these hormones, along with insulin and/or leptin, are associated with body weight retention in the year following childbirth. A secondary purpose is to explore whether these hormones are acutely associated with appetite and food intake in an* ad libitum* meal. Based on the foregoing, it is plausible to hypothesize that fasting or meal-induced concentrations of these hormones are altered during lactation and are associated with body weight retention. In particular, variations of one or all of these hormones could help explain the highly variable differences in body weight and body composition noted in lactating women [14]. In agreement with this premise, Cummings et al. [25] proposed that ghrelin is a thrifty gene product that evolved to help animals consume and store fat well, thereby increasing survival during times of reduced food availability or famine. It could be argued that lactation is a time when thrifty metabolism, acting to preserve maternal adiposity, might offer a survival advantage for both mother and offspring. Alternately, elevated ghrelin and/or lowered PYY or GLP-1 during fasting may serve as maternal signals to increase food intake to support the energy demands of lactation.

## 2. Methods

Twenty-four healthy, primiparous, postpartum, exclusively breastfeeding women and 20 never-pregnant controls were recruited from November 2009 through April 2013 and followed for one year. Women were recruited during late pregnancy and early postpartum using recruitment flyers distributed to clinics, physician offices, lactation consultants, and area hospitals. To qualify, participants had to be at least 18 yr of age, nonsmokers in good general health, and with a hemoglobin and thyroid stimulating hormone (TSH) concentration within the normal range (hemoglobin = 12.0–18.0 g/dL; TSH = 0.35–5.50 *μ*U/mL). Participants were excluded if they had renal, hepatic, endocrine, gastrointestinal, pulmonary, cardiac, or hematologic disease, including elevated blood pressure (>140/90 mm Hg); showed signs of depression, anxiety, disordered eating, alcoholism, or other psychological or substance abuse issues; or used prescription or over-the-counter medications/herbal preparations that could influence metabolism. Lactating participants were excluded if they had complications of pregnancy (e.g., gestational diabetes or pregnancy-induced hypertension), had a multiple birth, or either were not currently exclusively breastfeeding or did not intend to breastfeed for at least a year. Women were included regardless of delivery method as long as they had no activity restrictions at 4-5 wk postpartum. The never-pregnant controls were of an age and BMI range that allowed matching to the postpartum lactating group at baseline (4-5 weeks postpartum) for age, BMI, and race/ethnicity; this was accomplished by recruiting groups of control women following the baseline visits of groups of postpartum participants. Never-pregnant control women were excluded if they planned on becoming pregnant or relocating from the area within the year following their baseline visit. The study was approved by the Institutional Review Board at the University of Wyoming. Volunteers were fully informed of possible risks during all procedures before providing written informed consent.

### 2.1. Overview of Baseline and 6-Month and 12-Month Visits

**Figure 1:**
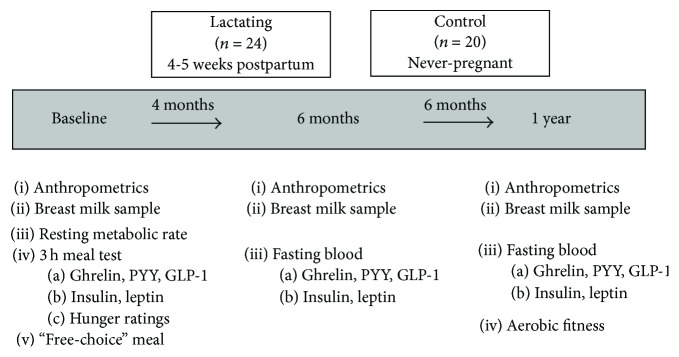
Schematic representation of the cross-sectional and longitudinal experimental design.

Lactating women were studied 4–6 weeks after delivery and at 6 and 12 months postpartum (Figure 1). Control women were initially evaluated and followed up at 5 and 11 months from their baseline date. At the initial visit, body weight, body composition, and resting metabolic rate (RMR) were measured and a meal test was performed to measure the hormonal (ghrelin, PYY, GLP-1, and insulin) responses to a standardized breakfast meal and* ad libitum* lunch. These same measures excluding the RMR and the meal testing were performed at follow-up (Figure 1). Blood was drawn after a 12-hour overnight fast for analysis of ghrelin, PYY, GLP-1, insulin, and leptin. Test days were scheduled during the follicular phase of the menstrual cycle for controls and lactating women who had resumed menstruation; serum progesterone concentrations were measured in all women to confirm status (i.e., serum progesterone < 2.0 ng/mL). Body composition was assessed by dual energy X-ray absorptiometry (Lunar Prodigy, GE Healthcare, Fairfield, CT) and waist circumference was measured at the level of the umbilicus.

#### 2.1.1. Baseline Testing

Food intake was controlled for 24 hours prior to baseline testing by providing a controlled diet which consisted of commercially available foods/beverages. Energy content of the diet was estimated using the equation of Redman et al. [26] based on body weight, age, and sex with an estimate of 1900 kJ (454 kcal) [27] added for lactating women. Participants were asked to consume all food/beverages provided and to return empty wrappers and any unconsumed food/beverages. Two snack bars (28.4 g, ~418 kJ; Clif Bar & Company, Berkeley, CA) were given as optional snacks above calculated energy requirements if the participant felt in need of additional energy. Participants were allowed plain water* ad libitum* but were asked not to consume other beverage or food not provided by the researchers. Participants were also asked to refrain from moderate-to-strenuous exercise for 24 hours before each test day.

On the test day morning, participants reported to the laboratory at 7:00 AM following a 12-hour overnight fast. Height and weight were determined using standardized procedures. RMR and fasting respiratory quotient (RQ) were measured over a 30 min period by indirect calorimetry using a commercially available metabolic cart (ParvoMedics TrueOne 2400, Sandy, Utah), calibrated before each test with standardized gases [28].

#### 2.1.2. Blood Draws

Immediately following the RMR, an intravenous catheter was inserted into a forearm or hand vein and connected to a sterile saline solution (0.9% sodium chloride) that was slowly infused (~30 cc/hour) to keep the catheter patent. After the baseline blood draw, participants were provided with a breakfast smoothie (15% protein, 60% carbohydrate, and 25% fat) that they were to consume in 15 minutes. The energy content of the test meal was individualized to provide 20% of each subject's estimated total energy expenditure (~1675–2500 kJ depending on weight, age, and lactation status). Blood samples were drawn 30, 60, 90, 120, and 150 minutes after test meal ingestion and 60 minutes following the* ad libitum* lunch meal (see below).

#### 2.1.3. Food Intake

*Ad libitum* food intake was used as a marker of appetite (commonly defined as the desire to eat) [29] and was assessed following the 150-minute blood draw.* Ad libitum* intake was determined individually in a room with the following food/beverages, in gram-weight portions, arranged on the table: ice water, cooked pasta (160 g dry), marinara sauce (140 g), Alfredo sauce (140 g), parmesan cheese, meat balls (3), hard-boiled eggs (2), whole wheat bread (2 slices), white bread (2 slices), fresh apples and oranges, milk, fruited-yogurt (3, individual containers), and individual portions of margarine, assorted-jellies, honey, and peanut butter. Subjects were told the meal was provided to allow baseline ratings for hunger and satiety on a full stomach and were instructed to eat as much as desired within 20 minutes [29]. Subjects were not allowed to read or study during the meal or carry bags/backpacks or coats into the room. The amount of food and fluid consumed was determined by weighing food remaining in the buffet as well as unconsumed food on the subjects' plates [30]. Protein, fat, carbohydrate, and total energy were calculated using a food analysis program (NutriBase 8 Professional Edition v.8.3.6) based on the USDA national nutrient database.

#### 2.1.4. Appetite Ratings

Hunger and satiety were assessed at baseline and at 30, 60, 90, 120, and 150 minutes after test meal ingestion (in accordance with blood draws) and at 20 and 60 minutes following the* ad libitum* meal using standard visual analogue scales (VAS). The questions included the following. (1) How hungry do you feel? (2) How satisfied do you feel? (3) How full do you feel? (4) How much do you think you can eat? The VAS was 100 mm in length and was anchored at each end by words describing the extremes of the appetite being measured.

#### 2.1.5. Hormone Analysis

Blood samples were collected, placed on ice, and cold centrifuged (2–8°C) to prevent protein degradation. For the gut peptides, 150 *μ*L of aprotinin and 40 *μ*L of DPP-IV were added to 4 mL EDTA-containing tubes immediately after collection for analysis of PYY and GLP-1 concentrations. Blood for analysis of ghrelin and its specific active or acylated form (ghrelin_acyl_) was collected into prechilled tubes containing 100 *μ*L of 200 mM AEBSF in addition to EDTA. Following separation of plasma, 200 *μ*L of 1 N hydrochloric acid was added for each 1 mL of plasma for ghrelin analysis only. Blood was analyzed in three batches near study completion. Leptin, total ghrelin, ghrelin_acyl_, PYY, and GLP-1 were analyzed by RIA using commercially available kits (Millipore Corp., Billerica, MA, USA) specific for humans as previously described [28]. Insulin analysis was also completed using a commercially available kit (Siemens Diagnostics, Tarrytown, NY). Fasting blood drawn at baseline was analyzed for leptin, progesterone, and prolactin as specified by the kit manufacturer (Progesterone, Siemens Diagnostics; Prolactin, Cayman Chemical Company, Ann Arbor, MI). Intra-assay CVs are as follows: leptin 8.1%, insulin 12.1%, ghrelin 4.4%, ghrelin_acyl_ 11.8%, PYY 10.1%, GLP-1 6.5%, and prolactin 2.3%. Interassay CV for assays analyzed in multiple batches was as follows: leptin 16.4%, ghrelin 9.7%, ghrelin_acyl_ 23.5%, PYY 10.5%, and GLP-1 17.9%.

#### 2.1.6. Longitudinal Visits

During the 6- and 12-month follow-up visits, participants reported to the laboratory between 7:00 and 9:00 AM following a 12 h fast. Height, weight, and body composition were measured and blood was obtained for analysis of fasting concentrations of leptin, insulin, ghrelin_acyl_, total ghrelin, PYY, GLP-1, and progesterone.

#### 2.1.7. Weight History and Menstrual and Lactation Status

Information on prepregnancy body weight, weight gain during pregnancy, infant's birth weight, and lactation status was collected from lactating women [31], and information on menstrual status was collected from all women via questionnaire and logs. Participants were provided logs to document lactation status and menstrual function which were initially provided at baseline and returned at the 6- and 12-month visits.

#### 2.1.8. Sample Size Determination

15 to 20 subjects per group were initially selected based on power/sample size calculations. Specifically, sample size calculations were performed using fasting and 60 min postprandial ghrelin from our previous study [23] which were the only data available at study initiation. These calculations determined that a sample size of 10 to 26 subjects would be required to detect (with 80–99% power) a difference between the postpartum lactating and nonpregnant control groups at either time point based on average concentrations in the lactating and control groups and a common standard deviation. Sample size calculations performed for the association between fasting ghrelin and fasting PYY and the change in body fat over the one-year follow-up period suggested 14 to 29 postpartum subjects would be required to detect (with 80–99% power) an association between fasting postpartum ghrelin and change in body fat if the true change in body fat mass is 0.007 kg per pg/mL change in fasting ghrelin [23]; the standard deviation of fasting ghrelin is 39 pg/mL and the standard deviation of body fat change is 2.0 kg.

#### 2.1.9. Statistical Analysis

At baseline, independent sample *t*-tests were used to test for differences in body weight/composition, food intake, and fasting hormone concentrations between lactating and control women. Changes in hormone concentrations and appetite ratings in response to the standardized meal were analyzed using ANOVA to test for a group (lactating, control) by time interaction or main effects for time or group. Data collected in response to the* ad libitum* lunch meal were not included in these analyses. Area under the curve (AUC) was calculated using the trapezoidal method corrected for baseline values (Prism version 6.05 for windows, GraphPad Software, Inc., La Jolla, CA) for appetite ratings and hormones that changed with meal ingestion including insulin, total ghrelin, ghrelin_acyl_, PYY, GLP-1, and the appetite ratings. For hormones and appetite ratings that were initially decreased after meal, including total ghrelin, ghrelin_acyl_ hunger, and desire to eat, both AUCs below (negative) and above (positive) were calculated. The effect of group for the AUCs was determined using independent sample *t*-tests. Separate ANOVA models were used to test for appetite and hormone responses to the* ad libitum* lunch.

For the longitudinal analysis, changes in body weight, body composition, and the fasting hormones over the 12-month period were analyzed by repeated measures ANOVA to test for a group by time (baseline, 6 months, and 12 months) interaction or main effects of time or group and by comparing the deltas of key variables (i.e., delta body weight from baseline to 6 and 12 months) using independent sample *t*-tests. Weight retention was analyzed similarly using delta body weight from reported prepregnancy weight to weight at 6 and 12 months. Standard correlation and ANOVA procedures were used to compare differences between women who lost within ±2 kg of prepregnancy weight versus those who retained weight. All statistics were computed using IBM SPSS Statistics version 22 (IBM Corporation). Data are reported as means ± SD. Significance was set at *p* < 0.05.

## 3. Results

### 3.1. Baseline Data

#### 3.1.1. Anthropometric Data

Physical and anthropometric characteristics of the postpartum lactating and never-pregnant control women are summarized in Table 1. Of the 24 enrolled, 8 were overweight prior to pregnancy (BMI > 24.9 kg/m^2^) and 16 were normal weight (BMI = 18.5–24.5 kg/m^2^). Lactating women gained 16.1 ± 4.1 kg during pregnancy, based on reported prepregnancy weight, and were studied at 5.0 ± 0.7 weeks postpartum. The never-pregnant controls were studied on day 5.3 ± 3.4 of their menstrual cycle and reported being weight stable for at least the past year. Lactating and control women were matched at baseline for age, height, weight, BMI, and body composition (Table 1). Lactating women, however, had larger waist and hip circumferences than control women (*p* < 0.001). All women had progesterone concentrations <2.0 ng/mL during baseline study, indicative anovulation or follicular phase of the menstrual cycle.

#### 3.1.2. Missing Samples

Baseline hip and waist circumferences are missing for one lactating subject and resting metabolism data are missing for two lactating and three control participants due to equipment failure or scheduling conflicts. Complete blood and hormonal concentration data were also not available for one lactating woman at baseline due to problems with catheter insertion during the meal test. In addition, occasional samples are missing for two lactating and two control women for ghrelin and ghrelin_acyl_, one lactating and one control participant for PYY, and two controls and five lactating for GLP-1 due to problems with indwelling catheter clotting at specific time points. Approximately 85% of missing samples occurred at 150 minutes after meal. Missing blood data that occurred at 60, 90, or 120 min were filled in by using the average of previous and subsequent points. Missing blood data at baseline, 30 min (after meal), or 150 min (immediately before* ad libitum* meal) however was considered missing; participants with missing data at these time points were not included in the repeated measures analyses and AUC analysis.

#### 3.1.3. Dietary Control

Energy and macronutrient intakes of the controlled diet averaged 9514 ± 2192 kJ (2274 ± 524 kcal; 16.1 ± 1.3% protein, 23.8 ± 3.4% fat, and 62.6 ± 5.2% carbohydrate) in the lactating and 9799 ± 1494 kJ (2342 ± 357 kcal; 15.3 ± 1.1% protein, 23.4 ± 2.5%  fat, and 64.3 ± 4.0% carbohydrate) in the control groups (*p* > 0.05).

#### 3.1.4. Resting Metabolic Rate

The RMR and resting RQ of lactating and control women are summarized in Table 1. No differences were detected between groups (*p* > 0.05) even after adjusting for differences in fat-free mass, fat mass, and age.

#### 3.1.5. Fasting Hormone Concentrations and Associations with Body Mass/Composition

Baseline concentrations of key hormones are summarized in Table 2. TSH was within normal limits for all women but was significantly lower in lactating compared to control women (*p* = 0.05). Prolactin concentration was also higher in lactating compared to control women (*p* = 0.001). Fasting insulin, leptin, ghrelin, ghrelin_acyl_, PYY, and GLP-1 concentrations, however, did not differ (*p* > 0.05) between groups. As summarized in Table 3, fasting leptin and insulin concentrations were associated with total body weight and body adiposity but not lean mass, whereas fasting ghrelin tended to correlate negatively with body fat. These associations did not change after adjusting for group (data not shown). There were no associations between fasting concentrations of ghrelin_acyl_, PYY, and GLP-1 and body mass, fat mass, or fat-free mass.

#### 3.1.6. Meal-Induced Changes in Hormone Concentrations and Appetite

**Figure 2:**
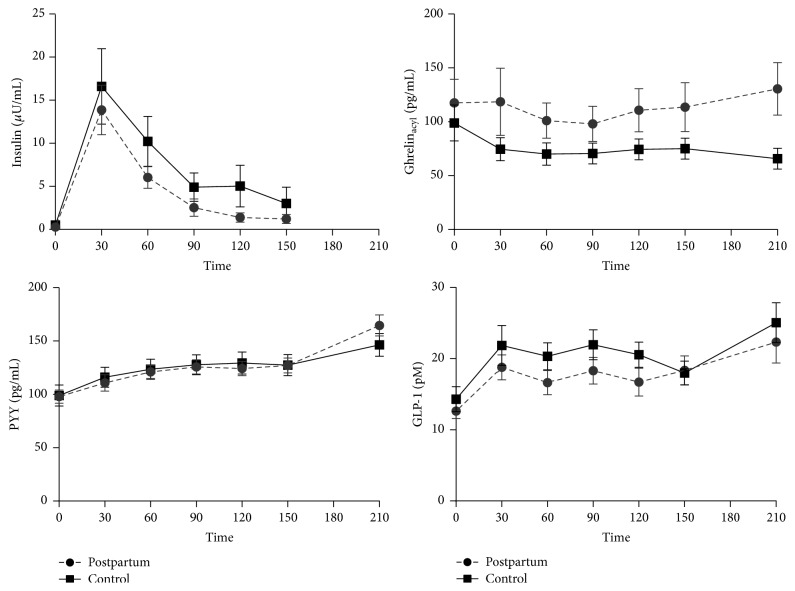
Fasting (time 0) and meal-induced responses of insulin, acylated ghrelin, PYY, and GLP-1 to a standardized breakfast meal in 24 postpartum lactating women (circles, dashed lines) and 20 never-pregnant controls (squares, solid lines).

The standardized breakfast meal provided 2087 ± 452 kJ (499 ± 108 kcal; 14.3 ± 1.1% protein, 22.9 ± 0.8% fat, 58.9 ± 0.5% carbohydrate, and 2.8 ± 0.7 grams of fiber) for lactating women and 1720 ± 134 kJ for control women (411 ± 32 kcal; 14.2 ± 0.4% protein, 22.9 ± 0.9% fat, 58.9 ± 0.8% carbohydrate, and 2.3 ± 0.4 grams of fiber). The insulin, ghrelin_acyl_, PYY, and GLP-1 responses to the standardized breakfast meal are shown in Figure 2 and AUC responses for all hormones presented in Table 2. Ghrelin (not shown) and ghrelin_acyl_ did not change significantly in response to meal ingestion (*p* > 0.05). The concentration of ghrelin_acyl_ was visibly higher in lactating versus control women after meal but there was only a trend for a group effect (*p* = 0.13 at 150 minutes). In contrast, insulin, PYY, and GLP-1 increased significantly (*p* < 0.001) following the standardized meal and fell over the next 150 minutes. These meal-induced responses, however, were not different between lactating and control women (*p* > 0.05, group *∗* time). Peak insulin responses (highest insulin concentration minus fasting insulin) were also not different between groups and averaged 19.6 ± 18.7 in the controls and 14.0 ± 13.3 *μ*U/mL (*p* = 0.27).

**Figure 3:**
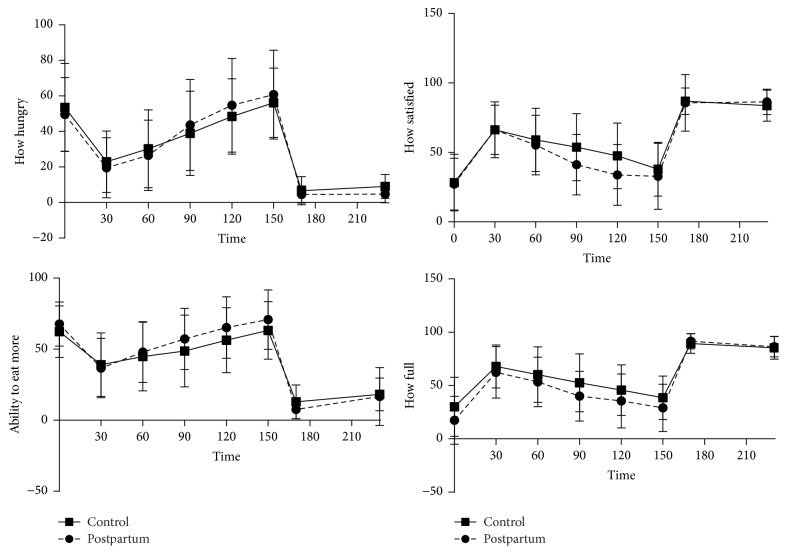
Ratings of hunger, satiety, fullness, and motivation to eat during fasting (time 0) and in response to a standardized breakfast meal in 24 postpartum lactating women (circles, dashed lines) and 20 never-pregnant control women (squares, solid lines).

Ratings of hunger and desire to eat decreased after the standardized breakfast meal and then increased over the 150 minutes postprandially in both groups (*p* < 0.05, time effect; Figure 3). Ratings of satiety and fullness demonstrated the opposite pattern. Despite small visual differences in appetite ratings between the lactating and control women between 60 and 120 min, no significant group differences were found (*p* > 0.10, group *∗* time and group effect). The AUC for the four appetite ratings was also not different between groups (*p* > 0.10).

#### 3.1.7. Ad Libitum Intake and Hormonal and Appetite Responses

Energy and macronutrient composition of the* ad libitum* meal varied considerably among individual participants. The lactating women consumed more energy (*p* < 0.05) (3678 ± 1146 kJ; 879 ± 274 kcal), protein (36.9 ± 10.8 grams), and carbohydrate (134.7 ± 47.6 grams) than the controls (2971 ± 816 kJ; 710 ± 195 kcal, 29.8 ± 10.0 g protein, and 108.6 ± 36.4 g carbohydrate) but the macronutrient composition and fiber content were similar (*p* > 0.05) between lactating (16.9 ± 2.2% protein, 24.6 ± 6.9% fat, 61.5 ± 8.4% carbohydrate, and 9.4 ± 3.5 g fiber) and control groups (16.6 ± 3.0% protein, 25.6 ± 7.2% fat, 61.0 ± 9.6% carbohydrate, and 7.6 ± 2.4 g fiber). The* ad libitum* meal resulted in increased (*p* < 0.05) serum concentrations of PYY, GLP-1, and insulin at 60 min postprandial compared to before initiation of the ad libitum meal (i.e., 150 minutes) (Figure 2). Hunger and desire to eat were decreased greater than those of the standardized breakfast and satiety and fullness were increased (Figure 3). Ghrelin and ghrelin_acyl_, however, did not change significantly over time (*p* > 0.05 for time) following consumption of the* ad libitum* lunch but plasma concentrations of ghrelin_acyl_ were greater (*p* = 0.04) at 60 minutes postprandially in lactating compared to control women (Figure 2).

#### 3.1.8. Prediction of Ad Libitum Intake

Energy intake was not predicted by concentrations of appetite-regulating hormones or hunger or satiety ratings prior to the* ad libitum* meal or by hormone response to the previous standardized meal. However, higher serum concentrations of ghrelin_acyl_ prior to the meal (at 150 min) and a higher ghrelin_acyl_ response (AUC) were associated with selection of more carbohydrate (*p* < 0.05, *r* = 0.33, and *r* = 0.39, resp.) and less protein (*p* < 0.05, *r* = −0.035, and *r* = −0.46) as a percent of total energy in the* ad libitum* lunch meal. A higher PYY AUC was correlated with higher absolute carbohydrate intake (*r* = 0.33; *p* < 0.05).

### 3.2. Longitudinal Data

Of the initial 24 lactating and 20 control women, 6 lactating and 5 controls were unable to complete the longitudinal measurements because of relocation, time constraints, or undisclosed reasons. Of the 18 lactating women, 9 continued to breastfeed for the duration of the study and 9 stopped lactating between baseline and the 6-month (*n* = 4) and 12-month follow-up (*n* = 5). The anthropometric characteristics of these 18 lactating and 15 controls are shown in Table 4. Anthropometric characteristics of the women lost to attrition did not differ (*p* > 0.05, data not shown) from those completing the study.

#### 3.2.1. Changes in Body Mass and Body Composition

**Figure 4:**
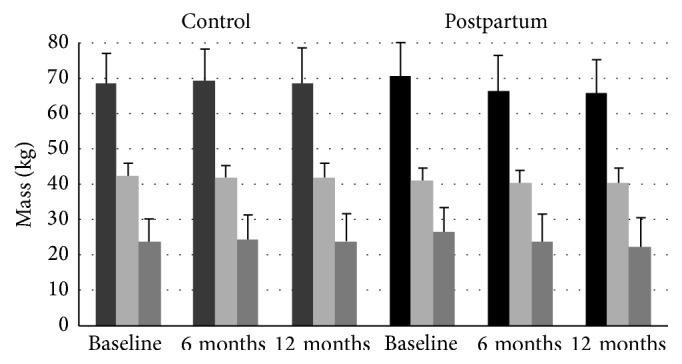
Total body mass (dark gray bars), lean mass (light gray bars), and fat mass (gray bars) at baseline and at 6- and 12-month follow-up in 18 lactating women and 15 never-pregnant control women.

During the follow-up period, lactating women lost 5.3 ± 2.2 kg of body mass whereas the body weight of control women remained stable (*p* = 0.02; group *∗* time interaction, Figure 4). Lactating women also experienced a reduction of fat mass, body fat percentage, and waist circumference (*p* = 0.011, *p* = 0.007, and *p* = 0.027, resp., group *∗* time interaction). Lean mass remained unchanged (*p* = 0.947) in both groups. The majority of loss in waist circumference, weight, and body fat occurred during the first six months postpartum (Figure 4; Table 2). At 12 months postpartum, 15 of the lactating women were within 2 kg of their prepregnancy weight while the remaining three were 6.0 to 17.5 kg heavier than prepregnancy weight. Change in total body mass and fat mass did not differ at either time point in the lactating women who continued to lactate at 12 months (*n* = 9) compared to those who discontinued lactation (*n* = 9) (*p* > 0.05).

#### 3.2.2. Change in Appetite-Regulating Hormones across the First Postpartum Year

Despite decreases in total body weight and adiposity in lactating women, fasting concentrations of leptin, ghrelin, ghrelin_acyl_, and GLP-1 remained relatively stable across the first postpartum year (Table 4). Fasting concentrations of insulin and PYY increased slightly in both lactating and control women (*p* < 0.05 for time).

#### 3.2.3. Association between Body Mass and Body Fat Loss and Appetite-Regulating Hormones

Absolute change in body mass and body fat for lactating women at either 6 or 12 months was not explained by concentrations of the appetite-regulating hormones at baseline either during fasting or in response to the standardized breakfast meal (AUC). Comparisons between women who returned to within 2 kg of their prepregnancy weight (*n* = 15) and those that had retained their pregnancy-associated weight gain (*n* = 3) suggested that those retaining weight had gained more during pregnancy (22.0 ± 5.7 versus 15.0 ± 3.4 kg, *p* < 0.05) despite similar self-reported [32] weight and BMI (63.2 ± 8.9 kg, 25.2 ± 3.0 kg/m^2^ versus 64.6 ± 8.5 kg, 22.6 ± 9.5 kg/m^2^) prior to pregnancy. The weight retainers also had higher fasting insulin during baseline testing (1.4 ± 0.8 versus 0.0 ± 0.0) and greater insulin (total AUC, 1299 ± 542 versus 446 ± 362, *p* < 0.05) and ghrelin rebound responses (i.e., higher response after the initial postmeal fall; positive AUC, 17155 ± 27648 versus 2893 ± 3033, *p* < 0.05) after the standardized breakfast meal.

## 4. Discussion

Failure to lose weight following pregnancy is an important and identifiable risk factor for long-term obesity [1, 4] and alterations in circulating fasting and meal-induced appetite regulators during lactation were considered as a possible mechanism of postpartum weight retention. We determined that fasting concentrations of these hormones and their postprandial pattern following standardized intake were not different between lactating and never-pregnant control women matched for age, body mass, and body composition. The only notable difference was the tendency for higher postprandial ghrelin_acyl_ in the lactating women (Figure 2) that was higher after the* ad libitum* lunch despite consumption of 24% more energy than control women. Circulating concentrations of appetite-regulating hormones were also not predictive of postpartum weight loss; however, higher postprandial ghrelin and insulin responses were observed in women who did not lose pregnancy-associated body mass by the end of the first postpartum year. This suggests that a greater response of the orexigenic hormone ghrelin may work against postpartum weight loss by either promoting increased appetite/food intake [33] and/or preventing body fat loss [32].

To our knowledge, this is the first study to comprehensively evaluate differences in fasting and meal-induced responses of the gut peptides ghrelin, PYY, and GLP-1 along with insulin and the adipose-derived hormone leptin. These hormones act in concert serving as signals between the gut, adipose tissue, and brain and are involved in appetite, glucose homeostasis, and body weight regulation [16–19]. Although the actions of these hormones are not completely elucidated, ghrelin is thought to enhance food intake through interactions with neuropeptide Y (NPY) and agouti-related protein- (AgRP-) expressing neurons of the hypothalamic arcuate nucleus whereas PYY, GLP-1, and leptin work via dampening these neurons [16, 18]. The role of these hormones in obesity pathogenesis is not yet delineated but peripheral infusion of ghrelin is shown to promote food intake in humans [33] and decrease fat oxidation and promote adiposity in animal models [32], whereas infusion of PYY and GLP dampens appetite and food intake in humans [34–36]. Our results suggest that differences in these hormones do not, however, predict intake in an* ad libitum* meal. Curiously, higher serum concentrations of ghrelin_acyl_ prior to the* ad libitum* meal and a higher ghrelin_acyl_ AUC were associated with selection of more carbohydrate and less protein.

Somewhat in contrast to the current results, our previous study found lactating and nonlactating postpartum women had lower fasting ghrelin concentrations compared to never-pregnant controls [23]. Our earlier study, however, measured only total ghrelin (not ghrelin_acyl_) and PYY at 60 min postprandially and was complicated by the higher body mass and adiposity in the postpartum women compared to leaner controls. The current study, which carefully matched lactating and control groups at baseline for body mass and body composition, also did not find differences in postprandial hormone concentrations apart from the consistently higher postprandial ghrelin_acyl_ in lactating women that was statistically different after lunch. Given the potential role of ghrelin, PYY, and GLP-1 in appetite regulation and energy balance, we had expected identifying a different fasting postprandial pattern in lactating women reflecting their increased energy demands. The higher ghrelin_acyl_ concentration in lactating women 60 min after consumption of the* ad libitum* lunch, despite consumption of 24% more energy, is the only notable finding and may suggest reduced suppression of hunger even after a full meal. This could promote shorter frequency of food intake initiation between meals. On the other hand, animal studies suggest this issue is not straightforward. Similar to our generally null findings, serum concentrations of ghrelin and PYY are also unaffected by lactation in rats and pigs [37–39]. However, higher circulating ghrelin concentration in lactating dairy cows is predictive of greater pasture intake during pasture grazing [40].

Another purpose of the current study was to evaluate whether varying concentrations or patterns of appetite-regulating hormones predict weight retention over the first year postpartum. This could help to explain the highly variable differences in body mass and body composition changes observed after childbirth and during lactation [14]. Butte and Hopkinson speculated over fifteen years ago that body composition changes during lactation are in response to a sequence of complex neuroendocrine and biochemical stimuli, many of which were unknown [14]. Although we did not find that fasting or meal-induced changes in gut peptides predict absolute body mass change, a greater ghrelin rebound after meal was observed in the three women who retained postpartum weight at the end of the first postpartum year. A higher fasting insulin and greater insulinemic response was further noted and is likely due to the higher adiposity of these women. Altered ghrelin may work to promote food intake and/or dampen fat oxidation preventing spontaneous body weight loss [32]. Although additional research is needed, there are practical implications. Women who increase body mass excessively during the first pregnancy or retained weight after delivery also have a higher risk in subsequent pregnancies of overweight and obesity [5]. These women should be targeted to receive appropriate advice and support.

Although well controlled, the current study was limited by sample size, use of a liquid versus a solid standardized meal, and the known general variability of both circulating concentrations of gut peptides among participants and the intra/intervariability of the assays. Even though our sample size of 24 lactating and 20 never-pregnant controls is relatively large given recruitment feasibility and well within the* a priori* target sample, it may have limited our ability to detect differences for various peptides—particularly ghrelin_acyl_ and GLP-1—and subjective appetite ratings. A larger and more diverse sample may have provided more statistical power to detect differences in the groups. Longitudinal data was likely limited by sampling from a healthy university community as well as the approximate 25 percent attrition and may have contributed to the small number of weight retainers at one year (3 versus 15). Our observation that weight retainers had higher ghrelin rebound responses (i.e., higher response after the initial postmeal fall), higher fasting insulin, and greater insulin meal after the standardized breakfast meal should be followed up in future studies. A final possible limitation was our decision to match controls based on body mass in early versus late postpartum. This may have masked true differences in the early postpartum period when lactating women are expected to be heavier than never-pregnant women.

## 5. Conclusion

Results of the current study are not in support of the hypothesis that appetite-regulating gut peptides and leptin are altered during lactation and associated with postpartum body weight retention. The potential for differences in the ghrelin_acyl_ and GLP-1 responses to a meal during lactation should be further evaluated in future larger-scale studies. Similarly, our observation that postpartum weight loss may be countered by alterations in postprandial ghrelin_acyl_ at early postpartum deserves further exploration in a larger, more diverse sample of women. Understanding how these and other appetite- and body weight-regulating hormones function during this particularly obesogenic stage of the women's lifecycle may be helpful in furthering the overall understanding of obesity pathogenesis.

## Figures and Tables

**Table 1:** Physical and anthropometric characteristics and resting metabolism in postpartum lactating and never-pregnant control women.

	Never-pregnant women (*n* = 20)	Lactating women (*n* = 24)	*p*
Race/ethnicity (W/L/A, MR)	18/1/1/0	21/1/1/1	
Age (yr)	25.7 ± 5.3	26.6 ± 6.1	NS
*Anthropometrics and body composition*			
Height (cm)	167.6 ± 5.9	166.7 ± 6.9	NS
Weight (kg)	68.7 ± 9.1	71.0 ± 10.0	NS
BMI (kg/m^2^)	24.0 ± 3.1	25.2 ± 3.6	NS
Body fat (%)	35.6 ± 7.6	39.4 ± 6.3	0.08
Fat mass (kg)	23.8 ± 7.8	27.1 ± 7.7	NS
Lean mass (kg)	41.9 ± 3.7	40.5 ± 3.8	NS
Body fat gynoid (%)	45.1 ± 6.2	48.4 ± 4.9	0.06
Body fat android (percentage)	37.8 ± 10.2	43.5 ± 8.4	0.05
Waist circumference (cm)	82.0 ± 9.0	93.2 ± 9.3^†^	0.000
Hip circumference (cm)	100.1 ± 7.9	103.4 ± 9.3^†^	NS
*Resting energy expenditure and sleep*			
RMR (kJ/day)	6263 ± 1063^†^	5812 ± 1192^†^	NS
Adj^†^ RMR (kJ/day)	6113 ± 506	5925 ± 490	NS
RQ	0.72 ± 0.05^†^	0.73 ± 0.06^†^	NS
Heart rate (bpm)	60.1 ± 39.2^†^	70.6 ± 25.7	

W, white; L, Latino; A, Asian; MR, mixed race; BMI, body mass index; RMR, resting metabolic rate; RQ, respiratory quotient; ^†^missing data; ^†^RMR adjusted for differences in fat-free mass, fat mass, and age (5925 ± 490 kJ/day, lactating versus control).

**Table 2:** Appetite-regulating hormones and other hormones during fasting and in response to a standardized meal.

	Never-pregnant controls (*n* = 20)	Lactating women (*n* = 24)	*p*
*Fasting*			
Prolactin (ng/mL)	19.0 ± 7.0	53.1 ± 30.2	0.0001
Thyroid stimulating hormone	2.2 ± 1.4	1.4 ± 0.7	0.05
Leptin (ng/mL)	16.4 ± 10.9	16.7 ± 9.9	NS
Insulin (*μ*U/mL)	0.51 ± 1.60	0.26 ± 0.68	NS
Ghrelin (pg/mL)	875.5 ± 333.6	763.3 ± 215.3	NS
Ghrelin_acyl_ (pg/mL)	98.9 ± 72.5	117.6 ± 102.8	NS
PYY (pg/mL)	99.0 ± 43.2	98.0 ± 30.2	NS
GLP-1 (pM)	14.3 ± 7.6	12.6 ± 5.0	NS
*Meal-induced responses (area under the curve, AUC)*			
Insulin (AUC, *μ*U/mL)	718 ± 625	1133 ± 1212	NS
Ghrelin (+AUC, pg/mL)	20947 ± 26658	13206 ± 12060	NS
Ghrelin (−AUC, pg/mL)	16620 ± 28432	6655 ± 6205	NS
Ghrelin (+AUC, pg/mL)	4339 ± 5722	6362 ± 11017	NS
Ghrelin_acyl_ (−AUC, pg/mL)	232.4 ± 393.2	1500.5 ± 3494.7	NS
Ghrelin_acyl_ (+AUC, pg/mL)	3923.4 ± 7548.0	2942.9 ± 5358.9	NS
PYY (AUC, pg/mL)	3378 ± 2201	3679 ± 3044	NS
GLP-1 (AUC, pM)	1017 ± 714	882 ± 874	NS

Ghrelin_acyl_, acylated (active) ghrelin; +AUC, standard AUC or AUC above baseline; −AUC, area below baseline.

**Table 3:** Association between body weight and body composition and fasting hormone concentrations in 24 postpartum lactating and 20 never-pregnant women.

Hormone	Weight (kg)	BMI (kg/m^2^)	Fat mass (kg)	Lean mass (kg)	Body fat (%)
Leptin	0.65^*∗*^	0.82^*∗*^	0.76^*∗*^	0.11	0.71^*∗*^
Insulin	0.31^*∗*^	0.35^*∗*^	0.37^*∗*^	0.03	0.34^*∗*^
Ghrelin	−0.25	−0.29	−0.28	0.04	−0.31^*∗*^
Ghrelin_acyl_	0.11	0.05	0.13	0.03	0.10
PYY	−0.16	−0.14	−0.25	−0.04	−0.23
GLP-1	−0.11	−0.02	−0.04	−0.13	−0.03

Data are correlation coefficients (*r*) between body weight and markers of body composition and fasting hormone concentrations in blood; ^*∗*^ *p* < 0.05; ghrelin_acyl_, acylated (active) ghrelin.

Note: adjustment for group does not significantly alter correlation coefficients.

**Table 4:** Anthropometric characteristics and fasting hormone concentrations of postpartum lactating and never-pregnant control women completing the longitudinal study.

Characteristic	Never-pregnant women (*n* = 15)			Lactating women (*n* = 18)			*p*
	Baseline	6 months	12 months	Baseline	6 months	12 months	
Height (cm)	169.5 ± 5.5	169.0 ± 5.9	169.0 ± 5.9	168.1 ± 7.7	168.1 ± 7.8	168.1 ± 7.9	NS
Weight (kg)	68.6 ± 8.6	69.1 ± 9.0	68.7 ± 9.9	70.8 ± 9.2	66.5 ± 10.1	65.6 ± 9.5	*∗*
BMI (kg/m^2^)	24.2 ± 2.7	24.2 ± 2.9	24.1 ± 3.2	25.0 ± 3.4	23.6 ± 3.8	23.0 ± 3.8	*∗*
Body fat (%)	35.3 ± 5.7	36.0 ± 5.9	35.8 ± 6.2	38.9 ± 5.8	35.9 ± 7.1	34.5 ± 7.9	*∗*
Fat mass (kg)	23.6 ± 6.4	24.2 ± 6.8	23.9 ± 7.6	26.5 ± 6.9	23.5 ± 8.0	22.2 ± 8.2	*∗*
Lean mass (kg)	42.4 ± 3.3	42.1 ± 3.2	42.1 ± 3.6	40.7 ± 3.9	40.2 ± 3.6	40.3 ± 4.2	*∗*
Body fat gynoid (%)	45.2 ± 4.5	45.2 ± 4.5	45.3 ± 5.0	48.2 ± 4.6	45.5 ± 6.3	44.0 ± 6.6	*∗*
Body fat android (%)	37.8 ± 9.0	39.5 ± 9.0	38.2 ± 9.3	42.7 ± 8.0	39.3 ± 8.8	37.3 ± 10.4	*∗*
Waist circumference (cm)	81.7 ± 8.3	81.3 ± 8.4	79.0 ± 16.2	92.9 ± 7.6	82.4 ± 16.1	82.0 ± 9.1	*∗*
Hip circumference (cm)	99.6 ± 7.4	102.3 ± 5.3	98.4 ± 17.8	102.9 ± 8.2	97.2 ± 17.5	99.4 ± 8.4	NS

Prolactin (ng/mL)	18.0 ± 5.8	23.2 ± 4.3	26.6 ± 4.6	58.2 ± 5.6	30.0 ± 4.2	21.5 ± 4.4	*∗*
Leptin (ng/mL)	15.1 ± 10.0	15.6 ± 9.1	16.3 ± 8.7	14.1 ± 7.4	13.8 ± 8.7	13.2 ± 9.9	
Insulin (*μ*U/mL)	0.7 ± 1.9	0.9 ± 2.2	1.9 ± 3.6	0.2 ± 0.7	1.1 ± 1.3	1.9 ± 4.0	†
Ghrelin (pg/mL)	830.6 ± 358.8	1111.7 ± 812.0	693.9 ± 318.2	731.9 ± 242.4	750.2 ± 358.8	704.2 ± 301.8	
Ghrelin_acyl_ (pg/mL)	105.9 ± 83.4	133.7 ± 85.6	83.9 ± 67.8	101.6 ± 107.8	79.0 ± 56.5	79.2 ± 79.8	
PYY (pg/mL)	110.7 ± 41.4	126.6 ± 44.2	138.4 ± 38.2	101.0 ± 31.1	137.7 ± 43.7	140.9 ± 49.8	†
GLP-1 (pM)	14.8 ± 9.2	15.3 ± 9.0	16.1 ± 6.6	12.0 ± 5.9	12.4 ± 6.3	11.4 ± 4.0	

Ghrelin_acyl_, acylated (active) ghrelin; ^*∗*^group by time interaction, *p* < 0.05; ^†^time effect, *p* < 0.05.
